# A Feasibility Study of Automated Detection and Classification of Signals in Distributed Acoustic Sensing

**DOI:** 10.3390/s25175445

**Published:** 2025-09-02

**Authors:** Hasse B. Pedersen, Peder Heiselberg, Henning Heiselberg, Arnhold Simonsen, Kristian Aalling Sørensen

**Affiliations:** 1 DTU Security, National Space Institute, Technical University of Denmark, 2800 Kongens Lyngby, Denmarkhh@dtu.dk (H.H.);; 2 Tordenskjold ApS, 2830 Virum, Denmark; 3 Faroese Telecom, 188 Hoyvík, Faroe Islands; asi@net.fo

**Keywords:** distributed acoustic sensing, anomaly detection, principal component analysis, enhanced maritime awareness, classification, HDBSCAN, t-SNE

## Abstract

Distributed Acoustic Sensing (DAS) is an emerging technology in the maritime domain, enabling the use of existing fiber optic cables to detect acoustic signals in the marine environment. In this study, we present an automated signal detection and classification framework for DAS data that supports near-real-time processing. Using data from the SHEFA-2 cable between the Faroe and Shetland Islands, we develop a method to identify acoustic signals and generate both labeled and unlabeled datasets based on their spectral characteristics. Principal component analysis (PCA) is used to explore separability in the labeled data, and Hierarchical Density-Based Spatial Clustering of Applications with Noise (HDBSCAN) is applied to classify unlabeled data. Experimental validation using clustering metrics shows that with the full dataset, we can achieve a Davies–Bouldin Index of 0.828, a Silhouette Score of 0.124, and a Calinski–Harabasz Index of 189.8. The clustering quality degrades significantly when more than 20% of the labeled data is excluded, highlighting the importance of maintaining sufficient labeled samples for robust classification. Our results demonstrate the potential to distinguish between signal sources such as ships, vehicles, earthquakes, and possible cable damage, offering valuable insights for maritime monitoring and security.

## 1. Introduction

Distributed Acoustic Sensing (DAS) is a technology that transforms existing optical fiber cables into large-scale acoustic sensors. Monitoring capabilities using DAS has proven to be efficient in a range of applications. It has been used in monitoring railway health, earthquake detection, pipeline monitoring, ensuring perimeter security, traffic monitoring, and avalanche detection. These diverse applications highlight the broad capabilities of DAS, with methods for detecting, characterizing, and localizing various events continuously evolving as the technology advances and gains wider recognition [1,2,3,4,5,6,7]. In the maritime domain, DAS has shown promise in detecting marine life such as whales, as well as tracking vessel activity. Optical fiber cables already installed on the seafloor, originally for telecommunication purposes, can be repurposed to monitor acoustic signals in the ocean environment. This enables non-invasive, wide-area monitoring of ship traffic, marine mammal movements, and potentially even underwater seismic or anthropogenic activity. As research in this area grows, DAS is emerging as a valuable tool for enhancing maritime situational awareness, marine conservation, and underwater surveillance [8,9,10]. Traditional marine monitoring systems, such as hydrophone arrays, sonar, and satellite-based surveillance, suffer from key limitations. Hydrophones provide high-resolution data but require dedicated deployments and suffer from sparse spatial coverage. Sonar systems are active and energy-intensive, requiring the emission of acoustic pulses that not only consume significant amounts of power but can also interfere with marine life, particularly marine mammals sensitive to sound. Satellite surveillance, including the use of publicly available constellations such as the Copernicus Sentinel, is effective for monitoring the surface conditions and vessel traffic. However, it lacks the capability to capture underwater acoustic signals or reliably detect small, unregistered vessels, especially in cluttered or remote maritime environments. In contrast, DAS enables continuous, passive acoustic sensing up to 171 km along the cable using pre-existing infrastructure, reducing both the deployment costs and environmental impact [11]. Furthermore, DAS excels at multi-source signal discrimination in complex marine environments. Its high spatial resolution and dense virtual sensing points allow it to distinguish between overlapping acoustic events, such as multiple ships passing in proximity, or separate marine mammal calls from background noise. By recording the distributed strain along the fiber, DAS captures spatio-temporal signal patterns that are particularly well-suited for unsupervised classification and anomaly detection. This makes it a uniquely powerful tool for understanding diverse marine soundscapes, detecting stealthy or unregistered vessels, and monitoring regions vulnerable to environmental or geopolitical disruptions.

DAS generates massive volumes of data, often more than a terabyte per day, depending on acquisition parameters such as the sampling frequency and spatial resolution/decimation. This large data volume requires fast and reliable methods for detecting signals of interest without manual labeling. In addition, classifying different types of signals helps reduce unnecessary storage and provide insight into unknown sources. Traditionally, external data has been used to mitigate the need for manual labeling of a large amount of DAS data, for example, by correlating DAS signals with earthquake databases or Automatic Identification System (AIS) data.

The growing use of optical fiber cables as a sensor platform has also made them a vulnerable component of critical infrastructure. This vulnerability has been highlighted by the occurrence of five sabotage events since October 2023 in the Baltic Sea. Optical fiber cables connecting the Nordic and Baltic countries have been deliberately damaged with an increasing frequency [12]. Trawling accidents have also disrupted the connection between mainland Norway and Svalbard, as well as between the Faroe Islands and the Shetland Islands, highlighting the urgent need for better signal detection and classification [13,14].

This study investigates the feasibility of developing a fast and reliable anomaly detector to identify signals of interest in DAS data. Current DAS monitoring workflows often rely on manual labeling to identify events such as ship passages, whale vocalizations, or road traffic. These approaches are labor-intensive, difficult to scale, and lack the capability for real-time or automated classification across diverse signal types. We aim to construct a compact labeled dataset using external sources, such as earthquake catalogs, AIS data, and known vehicle movement patterns, to enable downstream classification of previously unknown signals. Our approach addresses a key gap in DAS processing by combining automatic change detection, spectral feature analysis, and unsupervised classification, all with minimal pre-processing. This enables the rapid construction of large datasets without manual labeling and supports identification of signals’ origins without the need for visual inspection or manual feature analysis. Unlike methods such as those presented in [15], which detect anomalies but do not differentiate between signal types, we adopt a Gaussian-based change detection framework similar to that in [16] but extend it by integrating spectral analysis and unsupervised clustering to support the interpretability and classification of unknown events. The labeled dataset we generate demonstrates that classification of such signals using spectral features is feasible and effective.

Section 2 describes the processing of DAS signals for anomaly detection (Kullback–Leibler divergence) and classification (principal component analysis (PCA), Hierarchical Density-Based Spatial Clustering of Seven Applications with Noise (HDBSCAN)). Section 3 evaluates the methods used, comparing the use of spectral signatures and clustering to identify unknown signals. Section 4 reviews the detection performance, challenges such as noise and the classification accuracy, and future improvements.

## 2. Materials and Methods

This section covers the use of DAS as a sensor platform for continuous monitoring, explaining the principles behind how strain data are generated. It outlines the methodology for automated signal detection and how a labeled dataset is constructed using, e.g., online earthquake databases for signal correlation. The methodology focuses on studying spectral signatures from different sources, aiming to classify unlabeled signals and highlight distinctive features.

### 2.1. Distributed Acoustic Sensing (DAS)

DAS uses existing optical fiber cables to monitor acoustic vibrations and environmental changes for various applications. Using an interrogator system at one end of the fiber, DAS can function as a passive sensor network. The interrogator emits a light pulse into the fiber, which interacts with small impurities within the cable. The impurities within the optical fiber give rise to various scattering mechanisms. The most dominant scattering mechanism is Rayleigh scattering, which is measured by the interrogator system used in this study, the OptoDAS from Alcatel Submarine Networks, Les Ulis, France [17]. Rayleigh scattering results in a phase shift of the backscattered light pulse, and this phase shift is directly proportional to the strain applied to the fiber. This is the basic principle that makes it possible to extract information about any strain applied to the cable. This phase change is defined as [18]

(1) $$\Delta \phi =\frac{2\pi }{\lambda_{0}}\Delta L_{0}\approx \frac{4\pi n_{g}L_{G}}{\lambda_{0}}\left(\frac{\Delta L_{0}}{L_{0}}\right)$$

where Δϕ is the phase change and $\left(\frac{\Delta L_{0}}{L_{0}}\right)$ is the relative change in the optical path length, typically caused by external acoustic disturbances. This relative change corresponds to the longitudinal strain along the fiber axis and is denoted as $\varepsilon_{x}$. In DAS, it is this strain, primarily due to axial (longitudinal) deformation, that induces measurable changes in the optical phase. $\frac{4\pi n_{g}L_{G}}{\lambda_{0}}$ is a constant determined by the system, with $n_{g}$ being the refractive index of the fiber cable, $L_{G}$ the gauge length, and $\lambda_{0}$ the wavelength of the emitted light pulse. The light pulse typically has a wavelength of 1550nm because this wavelength corresponds to the lowest amount of attenuation in the optic fiber cable and thus increases the total length of the cable in which the strain can be sensed.

**Figure 1:**
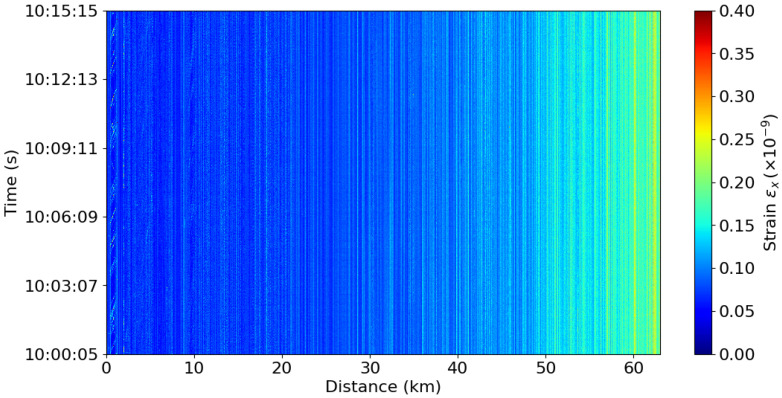
DAS data from the first 63km of the cable.

Figure 1 shows 15 min of DAS data, illustrating how the data typically appear in their normal representation, which is later used for signal detection. Although consistent signals are present in the first kilometer of the cable, corresponding to vehicles, this representation primarily reflects normal conditions without distinct signal events.

The persistent vertical white lines, visible along the cable throughout the figure, are caused by, e.g., environmental factors and faults in the cable that induce strain. These imperfections contribute to signal attenuation, as each interaction between scatterers and the emitted light pulse reduces the pulse’s energy.

#### Experimental Setup

**Figure 2:**
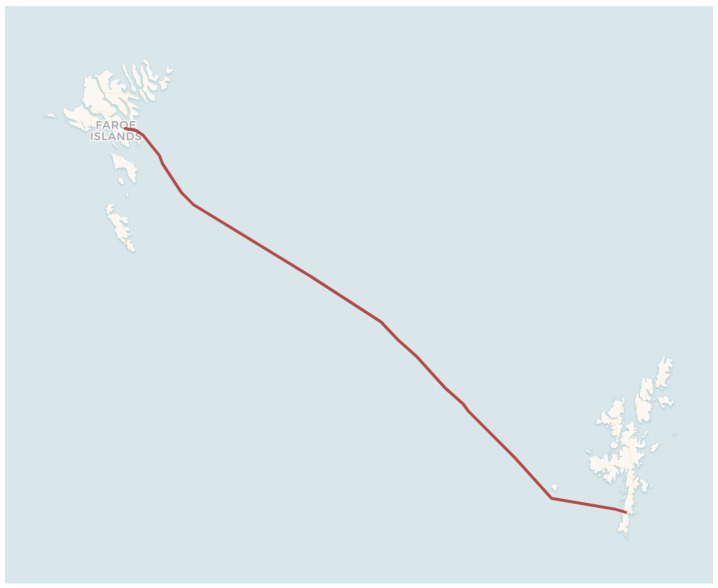
A map showing the SHEFA-2 optic fiber cable between the Faroe Islands and the Shetlands, used for data acquisition in this study. The interrogator was placed on the Faroe Islands.

The DAS data were acquired using the OptoDAS system from Alcatel along an optic fiber cable connecting the Faroe Islands with the Shetland Islands, illustrated in Figure 2. The fiber cable passes through areas that experience harsh environmental conditions, such as strong tides and winds, especially in the fjords near the Faroe Islands, inducing substantial noise in the DAS data [19].

The first kilometer of the cable is located on land before it extends into the ocean. The fiber cable section used for the data analysis was in shallow waters with depths ranging from 0 to 200 m. The cable consisted of both buried and unburied segments, depending on the location, and the seabed consisted of basalt and other rocky sediments. This resulted in a low signal-to-noise ratio (SNR) for the signals due to poor impedance matching in the coupling of the buried segments and the water in which the wave propagated [8].

The DAS system operated at a temporal sampling frequency of 800 Hz, allowing for the detection of signals up to 400 Hz, according to the Nyquist sampling theorem [20]. The OptoDAS system had a spatial resolution determined by the pulse width, *T*, of the frequency-swept laser pulse; $n_{g}$, the refractive index of the fiber; and c, the speed of light in vacuum: $\Delta x=\frac{c\cdot T}{2\cdot n_{g}}$. This yielded a spatial resolution for the OptoDAS system of 1.02 m. Spatial decimation was applied to reduce the storage demands by choosing only every fourth data channel. This yielded an effective resolution of 4.085 m. The gauge length was set to twice this resolution, 8.17 m, to preserve signals with small spatial footprints. The gauge length needed to be N≥2·Δx, where Δx is the spatial resolution and $N\in Z^{+}$. If necessary, this length could be extended further using a moving average filter to improve the SNR. Each data file generated with this configuration recorded data at 10 s intervals, with each file corresponding to 250 MB. The OptoDAS system generated snapshots of the raw DAS data in the spatio-temporal domain at 17 min intervals, allowing for the removal of unnecessary data, such as data for long periods with no visible signals. This approach helped optimize the storage by reducing the overall data volume, resulting in the storage of 5 terabytes of data.

### 2.2. DAS Signal Detection and Classification

We implemented an automated method to detect and classify signals in the DAS data, following a two-part process. First, the DAS data were used for signal detection. Some of the detected signals were annotated using earthquake databases, AIS data, and prior knowledge to label objects, e.g., vehicles, or semi permanent damage. The labeled data were then used for frequency analysis and PCA. HDBSCAN was used to classify unlabeled signals into separate clusters that were associated with the labeled data, and t-SNE was used for visualization.

#### 2.2.1. DAS Change Detection

The large data volumes generated by a DAS system are computationally demanding, as described in Section Experimental Setup. This often leads to manual identification of signals for data processing based on snapshots generated by the DAS system or by manually examining a large amount of data, sometimes aided by the use of external data sources, such as the AIS [8]. This issue highlights the need for a change detection algorithm that eliminates the need to manually identify signals. In addition, such an algorithm could capture small signals that are typically overlooked in manual labeling, since certain pre-processing steps may be needed to highlight these signals.

Change detection using DAS data can be approached in different ways. Our approach is based on trying to identify signals that significantly deviate from the normal representation of the data, typically caused by events such as cable damage or external disturbances like ships passing by. The primary objective was to develop a method that is computationally fast and efficient, enabling near-real-time processing while ensuring reliable detection. Further, the method is cable-agnostic, meaning that given a certain amount of observed data, this method works on any cable.

**Figure 3:**
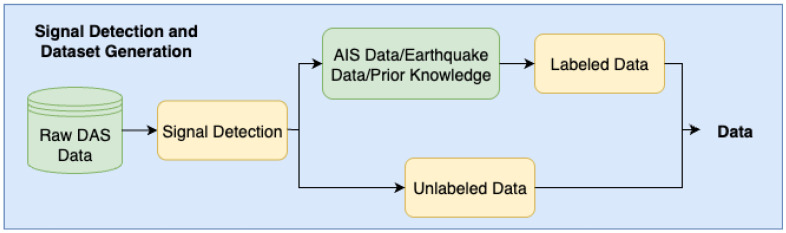
Flowchart of signal detection using the DAS data and how it is used to create labeled and unlabeled datasets for signal analysis and classification.

The process of detecting signals in the data and constructing a dataset based on labeled and unlabeled data is illustrated in Figure 3.

The main idea is to establish a normal representation of the strain data, when no signals are present—the raw strain data is used to prevent computational time being spent on pre-processing steps. This is achieved by analyzing several days’ worth of data and calculating the mean and variance for each data channel along the fiber cable. These values are used to construct a Gaussian distribution representing each data channel. When new and unseen data are processed, they are again treated as a Gaussian distribution, with the mean and variance calculated for a defined time window. The normal representation of the data does not explicitly account for tidal variations, as these are, to some extent, averaged during processing. However, this averaging was found not to hinder the detection of signals associated with changes in the tidal conditions. The change detection criterion is determined using the KL divergence, which measures the similarity between two distributions. This criterion was chosen because it accounts for variations in both the mean and variance of the data. Additionally, it is advantageous because it has a closed-form solution for two univariate Gaussian distributions. The KL divergence is defined as

(2) $$\mathrm{KL}\left[q||p\right]=\int_{x\in R}q\left(x\right)log\left(\frac{q(x)}{p(x)}\right)dx=\log\left(\frac{\sigma_{2}}{\sigma_{1}}\right)+\frac{\sigma_{1}^{2}+{\left(\mu_{1}-\mu_{2}\right)}^{2}}{2\sigma_{2}^{2}}-\frac{1}{2}$$

This measure is asymmetric, which means that KL[q||p]≠KL[p||q], which is why it is critical that q(x) represents the Gaussian distribution of the normal representation of the data and p(x) corresponds to the current data segment under evaluation. Swapping the two would yield different results and incorrect detection behavior. $\sigma_{1}$ and $\mu_{1}$ denote the variance and mean of q(x), and $\sigma_{2}$ and $\mu_{2}$ denotes the variance and mean of p(x). Equation (2) is minimized when the two distributions are identical, i.e., approaching zero. The divergence is computed over consecutive 2-second windows throughout the DAS time series. An empirically selected threshold of KL[q||p]≥1.1 was chosen to indicate the presence of anomalous signals. This threshold was determined by evaluating various values against a manually labeled subset of the data to maximize detection while minimizing false positives. The choice of this threshold is specific to the SHEFA-2 cable dataset, and it may need adjustment for other cables or deployment environments. Future improvements may include a formal optimization procedure such as Receiver Operating Characteristic (ROC) curve analysis to further refine the threshold selection.

Change detection is performed by loading 3 min of DAS data at a time. Spatial decimation is applied by selecting every fourth channel, corresponding to a spatial sampling interval of approximately 16 m, to reduce the computational time. This decimation factor was determined empirically by testing the algorithm on a signal with a small spatial footprint; the algorithm retained its ability to detect the signal reliably under this spatial decimation. Additionally, since nearby data channels are often correlated to some degree, this decimation results in minimal information loss.

A 3 min data window was chosen empirically to balance the loading time with a sufficiently large temporal window to capture entire signals. However, it is not always the case that the entire length of a signal is captured within a 3 min window. Signals, such as those generated by ships sailing along the cable, might be present for more than 3 min at a time. Here, AIS data were used to merge such signals. However, for signals originating from ships without AIS or sources without a ground truth, it is not possible to automatically merge these signals across 3 min windows. The 3 min data window is then divided into 2 s intervals for analysis using the detection algorithm. These intervals capture enough statistical data to approximate a Gaussian distribution while ensuring that small signals in the temporal domain are not averaged out, making them easier to detect. Using the KL divergence to detect signals results in a binary mask of areas where the signals deviate from the normal representation of the data, i.e., anomalies.

**Figure 4:**
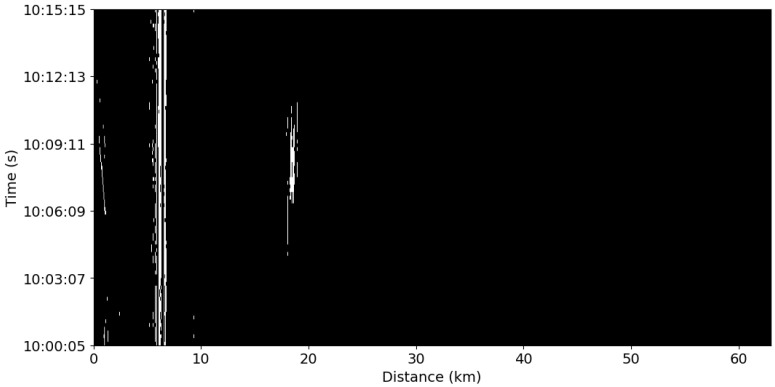
The binary mask generated by the change detection algorithm, showing 3 min of data, where the highlighted, bright areas correspond to anomalies. The binary mask highlights signals obtained over the first kilometer, corresponding to vehicles on a road. At around 6 km along the cable, a constant signal captured for the entire length of the window corresponds to semi permanent damage—strain induced by tidal changes. At around 19 km a signal is present for 90 s, corresponding to a ship passing near the cable.

A binary mask resulting from 3 min of DAS data is illustrated in Figure 4, highlighting the detection of different signals, such as those due to semi permanent damage at 6 km and vehicles at 1 km.

The anomalies detected in the binary masks are grouped into bounding boxes according to specific criteria for later analysis. Due to the noise inherent in the DAS data, the change detection algorithm may occasionally flag possibly irrelevant anomalies or miss genuine signals within the 2 s interval.

This is evident in Figure 4, which shows the detected signal at a distance of 19.6 km along the cable. The anomalies detected around the signal are not always coherent and may be dispersed, despite originating from the same source. To address these issues, the following criteria were established for creating bounding boxes around the anomalies detected using the binary mask:

- The signal must have a temporal length that is at least twice the sampling rate to ensure an adequate temporal resolution for spectral analysis.
- The signal must include data from a minimum of 20 data channels to ensure there is enough data for analysis.

It is important to note that the change detection algorithm can still detect weak disturbances and small events near the cable.

Based on the criteria for creating bounding boxes, it is possible for multiple bounding boxes to be generated from the same signal or for a signal to be split across two 3 min intervals when loading the data. Therefore, after the bounding boxes are created, additional criteria are applied to merge them.

- For two bounding boxes to be merged into a single box, their distance must be within 4 times the spatial resolution after decimation (65.36 m) of each other, and the time difference between them must be less than or equal to half the sampling period.
- Padding is added to the final bounding box to ensure the inclusion of signal edges that might fall below the threshold. This is particularly useful for cases where periodic signatures may exist at low frequencies but with weaker signal strength.

**Figure 5:**
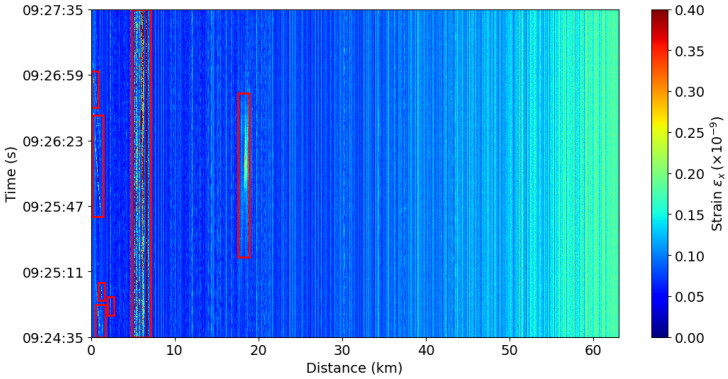
The generated bounding boxes based on the binary mask in Figure 4. They illustrate four different detected signals. From left to right, 5 boxes detect *vehicles*, 1 detects *semi permanent damage*, and 1 detects a *ship*.

The resulting bounding boxes, generated using the binary mask shown in Figure 4, are illustrated in Figure 5. These are generated based on empirically selected criteria optimized to detect two key signal types: *ship* signals and *semi-permanent damage*. Given the diversity of the signal characteristics in DAS data, it is challenging to define universal criteria for the generation of bounding boxes. Therefore, an empirical strategy was used in which various parameter configurations, such as the temporal and spatial thresholds, were tested. The final configuration was selected based on its ability to reliably isolate and group relevant signal regions, particularly for the two aforementioned signal types. To construct the dataset, we used a combination of labeled and unlabeled data, as outlined in Figure 3. Labeled signals were annotated by correlating bounding boxes with external database sources, including *AIS* data for vessels, seismic event catalogs, and known cable features (e.g., the *semi permanent damage* located at around 6 km along the cable, and vehicles passing on a nearby onshore road within the first kilometer). In this context, “prior knowledge” refers to a combination of expert interpretation, external data sources (e.g., AIS and seismic records), and historical observations of persistent or well-known signal patterns. Where manual labeling was necessary, a single expert annotator performed the annotations. The unlabeled dataset consisted of signals detected by the change detection algorithm that did not match any of the known signal types or available reference data and thus could not be confidently assigned a label. These segments were excluded from supervised training but were used afterwards in the classification to identify and interpret the unknown signals. A more detailed description of the dataset can be found in Section DAS Dataset.

A limitation of using the KL divergence is its assumption of Gaussian-distributed noise. If the noise instead follows a Poisson distribution, sometimes observed in optical systems, the closed-form KL divergence becomes less valid and may reduce the detection sensitivity. This could, in theory, affect the resolution by making it more difficult to detect weak or short-duration signals, as the effective SNR would be lowered and the noise statistics would deviate from Gaussian assumptions. Noise affects the resolution by limiting the ability of the system to distinguish between closely spaced or overlapping events and influences signal classification by altering the precision with which signals can be detected and separated from background noise. However, this was not observed in our data. The high temporal sampling rate of 800 Hz combined with the use of 2 s detection windows ensured that the noise characteristics closely approximated Gaussian behavior, thus preserving both the detection performance and resolution.

#### 2.2.2. DAS Signal Classification

**Figure 6:**
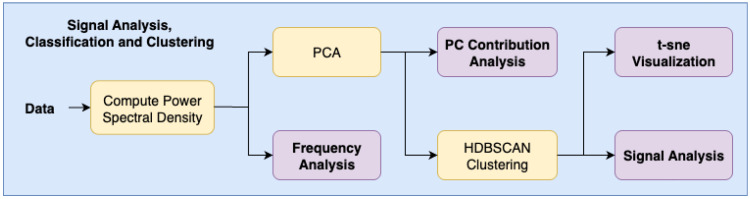
Flowchart of classification of signals using their spectral signature, PCA to reduce dimensionality, and HDBSCAN to cluster signals for classification.

After the anomalies had been detected, we determined the origin of the different signals using the labeled data. The classification of the signals was based on their spectral signatures. These signatures can be distinct for different types of sources or even different signals originating from the same type of source, e.g., ships. To analyze these signatures, the power spectral density (PSD) of each signal was computed, providing a frequency-domain representation of the signal’s power distribution and allowing for identification of its dominant frequency components. Frequency analysis was performed on the labeled data to capture their distinct spectral signatures. PCA was applied to the PSD of each signal to reduce the dimensionality, and HDBSCAN was used to cluster unlabeled signals by comparing them to clusters formed by the labeled data. The resulting clusters were visualized using the t-SNE algorithm. This process is illustrated in Figure 6.

To reduce the high dimensionality of the PSDs and facilitate visualization, PCA was applied [21]. The PCA decomposed the signals into [22]

(3) $$Cv_{p}=\lambda_{p}v_{p}$$

where **C** is the matrix of all the stacked PSDs for each detected signal. The dimensions of **C** are [S,N], where *S* is the total number of signals, *N* is the dimension of the frequency axis in the PSD, $\lambda_{p}$ represents the eigenvalues, and $v_{p}$ represents the corresponding eigenvectors. The contribution from each of the principal components was also investigated, to see what spectral features they captured. This was performed by projecting the eigenvectors, $v_{p}$, onto the PSDs of the signals [22].

(4) $$a_{mn}^{\left(p\right)}=\sum_{j=1}^{J}v_{p}^{\left(j\right)}d_{mn}^{\left(j\right)}$$

Here, *j* represents the *j*th frequency bin, $d_{mn}$ represents the magnitudes of the frequency bins in the PSDs, and $a_{mn}$ represents the projected coefficients. After applying PCA, the next step was to use the HDBSCAN clustering algorithm to group signals from different sources. HDBSCAN is an unsupervised machine learning algorithm that identifies clusters of varying shapes and densities in the data and is particularly useful when dealing with noise and outliers. It can help mitigate the impact of signals falsely labeled by the change detection algorithm by grouping signals with similar spectral features while distinguishing them from noise and irrelevant data [23]. Unlike traditional clustering methods such as the K-means, HDBSCAN does not require the number of clusters to be predefined. It works by building a hierarchy of clusters based on the density of the data points and then selecting the most stable clusters as the final output. The algorithm assigns points that do not belong to any cluster as noise, making it robust to any misclassified signals in the data and outliers in the unlabeled dataset. HDBSCAN is defined by [24]

(5) $$d_{\mathrm{mreach}-k}\left(a,b\right)=\max\left\{{\mathrm{core}}_{k}\left(a\right),{\mathrm{core}}_{k}\left(b\right),d\left(a,b\right)\right\}$$

where d(a,b) is the Euclidean distance measure, and ${\mathrm{core}}_{k}\left(a\right)$ and ${\mathrm{core}}_{k}\left(b\right)$ represent the core distances of points a and b, which are the distances to their *k*th nearest neighbors.

HDBSCAN therefore results in an unknown number of clusters, which may not align with the number of unique labels in the dataset. Using the labeled data, it can be possible to determine what each cluster represents. This allows for the potential determination of the source of unlabeled data if it is assigned to a specific cluster or even a new category of labels corresponding to a cluster that was not known to be in the data, e.g., mammalian life. In contrast, signals that are identified as outliers may not correspond to any specific label or cluster, meaning they might simply be noise.

## 3. Results

This study aimed to explore the feasibility of automatically detecting and classifying signals in DAS data. Building on existing research in DAS signal detection, the study developed a cable-agnostic methodology for automatic detection of signals along the cable. In short, the signal detection and classification methodology outlined in Section 2.2 used the KL divergence to identify signals. This was followed by principal component analysis and HDBSCAN clustering, with the results visualized using the t-SNE algorithm to classify unlabeled data.

### 3.1. Object Detection

**Figure 7:**
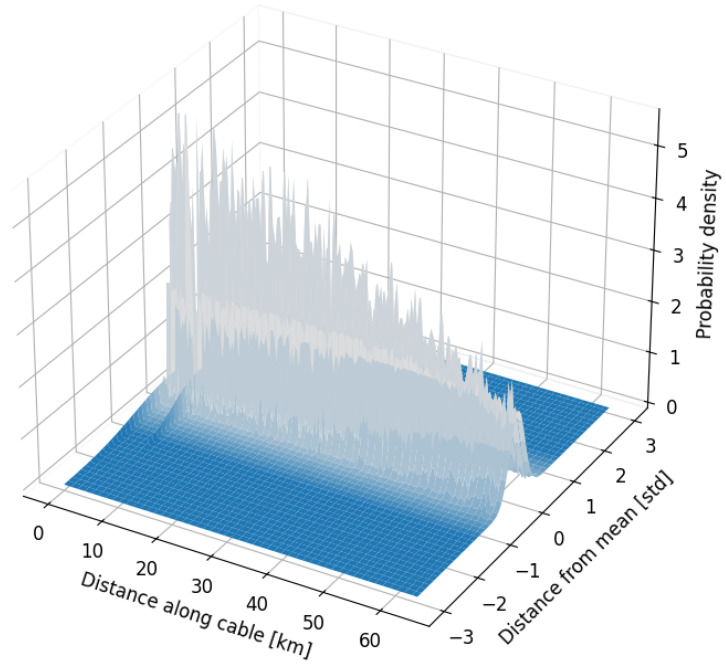
Normal representation of the different data channels for the first 63 km of the fiber optic cable used for data acquisition.

The object detection algorithm outlined in Section 2.2.1 created a normal representation of the data, reflecting the typical conditions over an extended period of time. This is illustrated in Figure 7, which shows the probability density function based on the mean and standard deviation of each data channel.

The figure clearly illustrates the attenuation of the signal as the distance from the interrogator increased. In the first segment of the cable, larger standard deviations appeared at 1 km and 6 km. At 1 km, this was due to the constant traffic of passing vehicles on land, which created greater variation in the data. At 6 km, the variation was attributed to shifting ocean tides, which pushed the cable against the rocky seabed, inducing permanent strain with low-frequency variations at that exact location. This phenomenon is also referred to as *semi permanent damage* in the labeled data. Using a representation of how the data normally looks, faults or areas of potential risk could be identified, providing an efficient method for preventing the loss of cable functionality.

Each channel in the normal data representation was compared with new, unseen data recorded at 3 min intervals. This data was further segmented into 2 s intervals, as described in Section 2.2. Anomalies were detected through this process, leading to the creation of bounding boxes around the signals, as illustrated in Figure 5. These bounding boxes were what was used to create the labeled and unlabeled datasets.

#### DAS Dataset

This dataset was constructed using the methods outlined in Section 2.2. A total of 1476 signals were detected over 30 days of data collection. The detector successfully identified signals from various sources, including *ships*, *vehicles*, *earthquakes*, and *semi permanent damage* to the fiber cable. Although the definition of *semi permanent damage* is somewhat vague, it is characterized by sustained strain over time— typically 8–12 h—caused by tidal changes. The detected signals comprised a small dataset with 164 labeled signals and 1312 unlabeled signals, as detailed in Table 1. Annotation was performed after all the detections were made and stored with their metadata, including the position along the cable, timestamp, area covered, and coordinates.

The *ship* signals were labeled by correlating the location of the detected signals along the cable with that of nearby ships using AIS data. A correlation threshold of 200 m was applied to account for potential inaccuracies in the cable’s exact placement, a known source of error when pinpointing signal origins, and errors in the interpolated AIS tracks.

The *earthquake* signals were labeled by cross-referencing online earthquake databases. The detected signals were then matched to earthquakes that occurred at the same time near the location of the fiber cable.

The *vehicle* signals were manually labeled based on prior knowledge of the cable’s placement on land, specifically within the first kilometer. The signals of vehicles moving on land exhibit distinct characteristics [6] and are easily identifiable.

Finally, *semi permanent damage* labels were assigned using prior knowledge of the cable conditions, as these can vary due to tidal effects that cause the cable to shift on the seabed in sections where the fiber cable is unburied.

**Figure 8:**
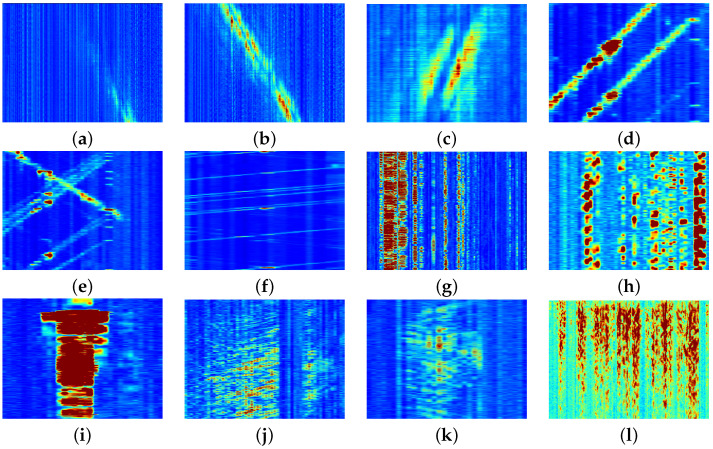
Examples of strain in the spatio-temporal domain: (**a**–**c**) show ships, where (**a**,**b**) show the same ship at two different time intervals, (**d**–**f**) show vehicles on land for the first kilometer of the cable, (**g**–**i**) show semi permanent damage of various extents, and (**j**–**l**) show earthquakes at different places along the fiber cable.

Figure 8 illustrates three examples from each labeled category in the dataset.

These examples were selected to highlight the variety of detections made by the detection algorithm and to demonstrate how the methodology can sometimes result in incomplete or excessive detections. For example, detections (a) and (b) represent two separate detections of the same ship. This occurred because of the methodology used by the detection algorithm, as described in Section 2.2.1, where data chunks were processed at 3 min intervals. By correlating these detections with AIS data, their bounding boxes could be combined into a single detection. Similarly, other signals could be combined if they met the criteria specified for merging bounding boxes. Another example is shown in Figure 8e–f, where multiple vehicles were detected simultaneously in each case. As a result, their signals combined into a single spectral signature during analysis, making it impossible to isolate the spectral characteristics of individual vehicles.

**Figure 9:**
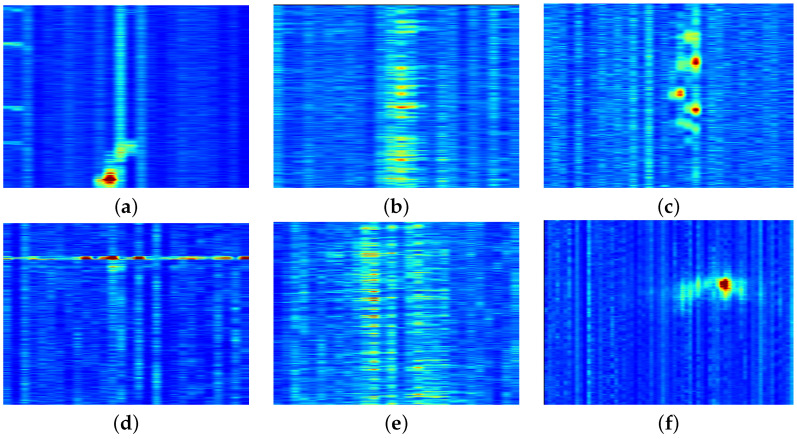
Examples of strain in the spatio-temporal domain: (**a**–**f**) show signals captured by the object detector corresponding to unlabeled data.

Figure 9 shows six examples of different types of signals we were unable to label based on the AIS, earthquake databases, and our prior knowledge. The figure illustrates the variety of ways in which signals can differ in their size and shape. When comparing them with the labeled data, no clear match emerges, suggesting that these signals may originate from sources not labeled in the data. This indicates the need for an analysis to classify these signals.

### 3.2. Object Classification

**Figure 10:**
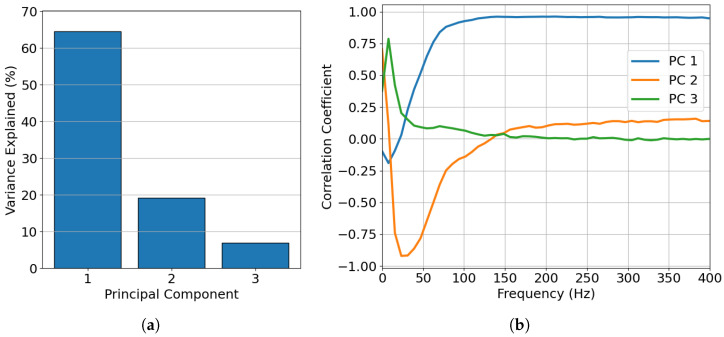
(**a**) shows the variance captured by the first three principal components. (**b**) illustrates which frequencies the first three principal components captured.

The clustering of signals was based on the spectral signatures of the signals, using a principal component analysis to reduce the dimensionality and extract the most important features. Analyzing the information captured by the different principal components and their dominant frequencies provides valuable insights into the results. As shown in Figure 10, the first three components accounted for 90% of the spectral information. The first component had a high correlation with signals with frequencies above 50 Hz and thus captured this range the most, meaning that it had difficulties separating spectral features above this frequency. The second component had a high negative correlation with signals centered at around 30 Hz and a positive correlation with low-frequency components. This means that the second component was good at discriminating between low frequencies and frequencies of around 30 Hz. The third component captured lower frequencies near 5 Hz. However, the principal components did not show variations at higher frequencies, meaning that it could be difficult to discriminate between high-frequency signals. We hypothesize that frequency components above 50 Hz do not contribute significantly to discrimination between different signal types, as most signals exhibit frequencies below the 50 Hz threshold, as will be demonstrated in subsequent analyzes. However, in cases where differentiation between signals originating from the same source is required and those signals exhibit dominant frequencies of above 50 Hz, it would be necessary to apply PCA specifically to signals from that source to enable effective discrimination between them.

**Figure 11:**
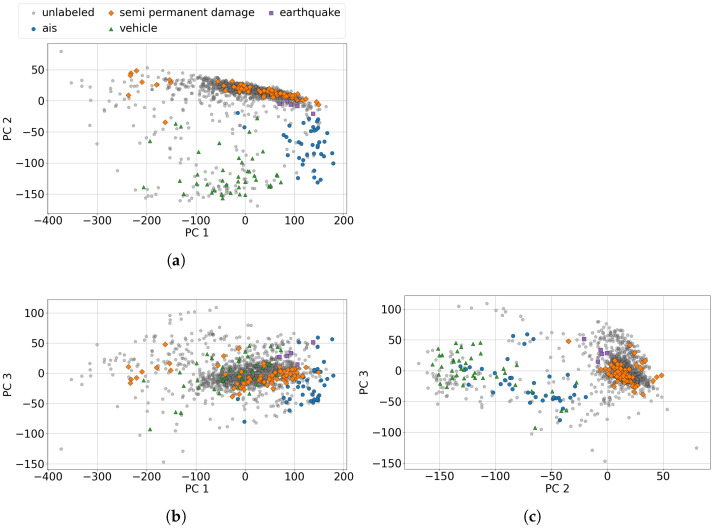
Principle component analysis of the power spectral densities of the labeled and unlabeled signals: (**a**) PC1 and PC2, (**b**) PC1 and PC3, and (**c**) PC2 and PC3.

**Figure 12:**
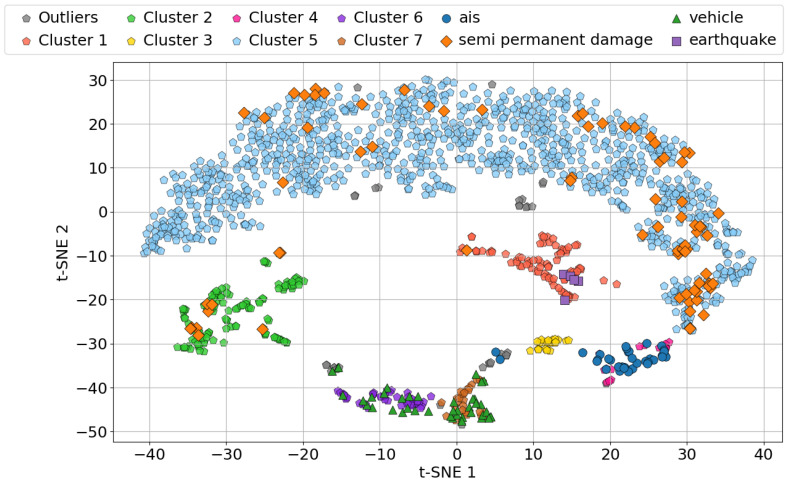
Clustering using the HDBSCAN algorithm on the first two principle components.

As well as applying principal component analysis to the labeled data for dimensionality reduction, the same transformation could be used to analyze the unlabeled data. Figure 11 illustrates the labeled and unlabeled data in two dimensions using the first three principal components. Figure 11a shows the first and second components, which correspond to the dominant signal frequencies of 30 Hz and above 50 Hz. It is clear that the majority of signals from *vehicles* and *ships* were negatively correlated with principal component 2, which means that they had dominant frequencies of around 30 Hz. The first component was centered at around zero for *semi permanent damage*, with variation, suggesting a dominant low-frequency signal for most of these signals. Figure 11b shows the first and third components, where most signals are difficult to distinguish. Both components were centered at around zero, with the first component exhibiting a slight positive trend and the third a negative trend. Figure 11c shows the second and third components, where *vehicles* and *ships* are clearly characterized by a higher frequency than *earthquakes* and *semi permanent damage*. This frequency difference separates the labeled data. Specifically, *vehicles* and *ships* exhibit dominant frequencies of around 30 Hz, while *earthquakes* and *semi permanent damage* are mainly associated with lower-frequency components. Figure 12 shows the clustering of the signals using the t-SNE algorithm. As shown in Figure 11, PCA performs a linear dimensionality reduction, whereas the t-SNE algorithm is a nonlinear method that preserves local structures. This nonlinearity can help clusters appear more well-defined and separated. By applying the HDBSCAN algorithm for classification using the first two t-SNE components for visualization, a clearer structure of the different clusters and their corresponding labels can be seen. The HDBSCAN algorithm was applied using the following parameters: a minimum cluster size = 20 and a minimum number of samples = 5. These values were empirically selected by evaluating multiple combinations of parameters and visually assessing their alignment with the labeled data. As the parameter selection was based on empirical visual assessment rather than quantitative optimization, this process may have introduced observer bias in the evaluation of the HDBSCAN performance. Accordingly, the clustering results should be interpreted with caution, acknowledging this methodological limitation.

Seven distinct clusters were identified, with some outliers present, signals that could not be associated with any of the clusters corresponding to the labeled data. Cluster 3 (yellow) was seen to be a cluster with no known signals associated with it but with spectral features similar to those of ships. What these signals might have originated from is up to speculation.

Most unlabeled data points were concentrated near the *semi permanent damage*-labeled signals, corresponding to cluster 5. However, a smaller subset associated with *semi permanent damage* was also identified as cluster 2. These two clusters arose because the *semi permanent damage* category is somewhat vague, encompassing both cable damage and strain induced by tidal changes in the section of unburied cable.

Two clusters, cluster 6 and 7, were identified as corresponding to *vehicle* signals. This differentiation was likely due to varying types of vehicles, such as motorbikes and cars. However, without further verification, this remains speculative.

*Earthquake* signals were predominantly associated with cluster 1, and a group of unlabeled signals was also concentrated in this region. This could suggest that earthquakes not present in the database used for correlation were detected through DAS, or alternatively, these signals could have been caused by another source with similar spectral characteristics.

*Ship* signals were associated with cluster 4, and some unlabeled signals were also associated with this cluster. This strongly indicates that potential *dark ships* were present in the data, emphasizing the effectiveness of identifying *dark ships* using DAS. Two signals with AIS data did not seem to correspond to any cluster and were between the clusters associated with *vehicles* and *ships*, alongside a few unknown signals. These signals were thought to have originated from ships that were trawling, adding a lower-frequency component to their spectral signature.

**Figure 13:**
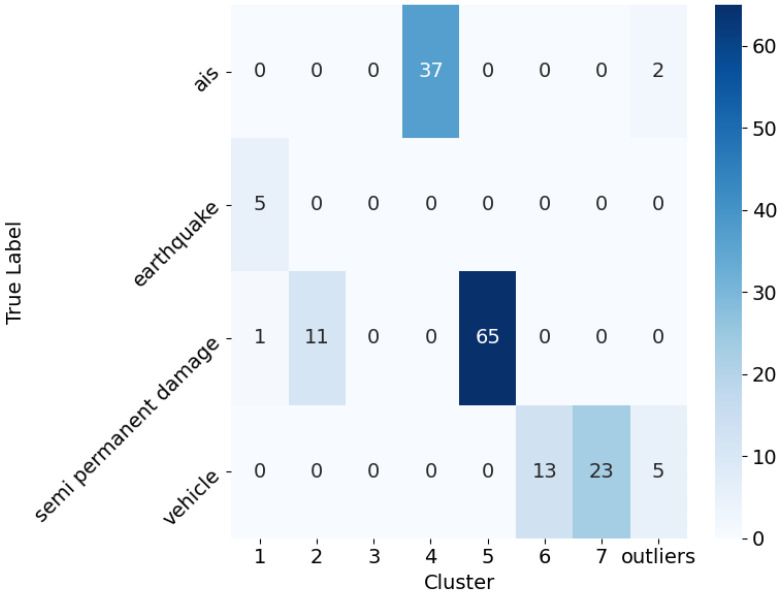
Confusion matrix for the labeled signal classification.

The confusion matrix of the classification based on the HDBSCAN results presented in Figure 12 can be seen in Figure 13, created using only the labeled data. To evaluate the performance of the clustering algorithm with respect to the known labels, we chose a commonly used metric: the F1 score.

This metric provides complementary insights into the quality of the clusters by combining the precision and recall into a single measure. The F1 score is defined as [25]

(6) $$F1\;\mathrm{Score}=2\cdot \frac{\mathrm{Precision}\cdot \mathrm{Recall}}{\mathrm{Precision}+\mathrm{Recall}}$$

where the precision and recall are defined as

(7) $$\mathrm{Precision}=\frac{TP}{TP+FP},\;\mathrm{Recall}=\frac{TP}{TP+FN}$$

Here, TP, FP, and FN denote the number of true positives, false positives, and false negatives, respectively. The precision reflects the proportion of correctly identified instances among all the instances assigned to a cluster, while the recall measures the proportion of the actual instances correctly identified by the clustering.

From the results in Table 2, we can observe that the clustering algorithm performed very well across all the labeled categories. The precision values were close to or equal to 1 for most labels, indicating that the clusters contained very few false positives. The recall values were slightly lower for some categories, particularly for *semi permanent damage* and *vehicles*, which suggests that a small fraction of the true instances were not assigned to the corresponding clusters (false negatives). The F1 score, which balances the precision and recall, confirmed these observations by being high for all the labels, with values ranging from approximately 0.91 to 0.97. This indicates that the clustering method reliably groups signals corresponding to each label, with a good balance between identifying true positives and avoiding false assignments.

The clustering results shown in Figure 12 were further evaluated using three metrics: the Davies–Bouldin Index [26], Silhouette Score [27], and Calinski–Harabasz Index [28]. These metrics were computed for both the full labeled dataset and for versions in which 10% increments of the data were systematically removed to observe how the quality of the clustering changed. The results are presented in Table 3. The Davies–Bouldin Index, which measures the similarity between clusters, generally increased as more data were dropped—from 0.828 with the full dataset to values of above 1.6 when 20–40% of the labeled data were excluded—indicating that the clusters became less distinct. The Silhouette Score, which ranges from −1 to 1, decreased from 0.124 with the complete dataset to negative values at the 20%, 30%, and 40% exclusion levels, suggesting that many data points became closer to neighboring clusters than to their own, a sign of degraded cluster cohesion and potential misclassification. The Calinski–Harabasz Index, which evaluates both the compactness and separation of groups, also decreased noticeably from 189.8 when using the complete dataset to approximately 120 in the 20–40% exclusion range, further confirming the deterioration in the quality of the grouping. These metrics clearly show that excessive removal of labeled data negatively affects the clustering performance. Maintaining a sufficient portion of labeled samples is therefore crucial to ensure the presence of meaningful and stable cluster structures in the dataset.

**Figure 14:**
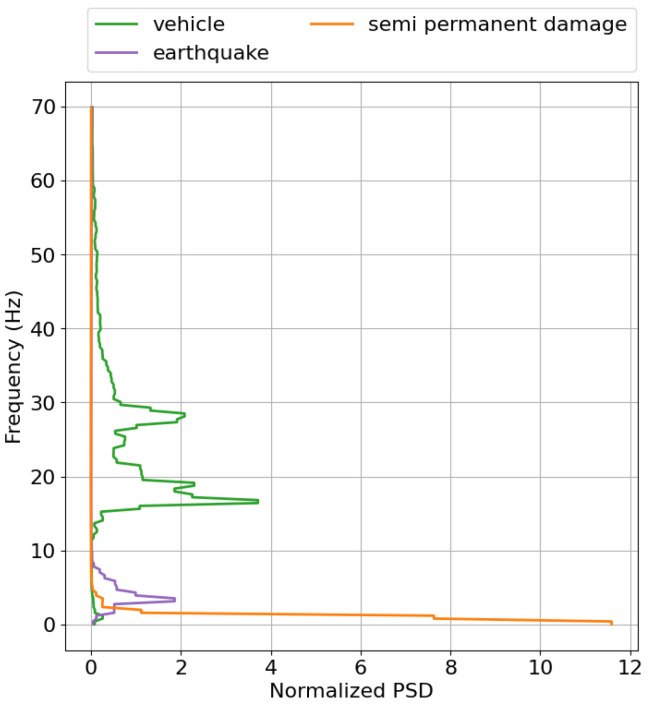
Dominant frequencies for the four different labels: *ship*, *vehicle*, *semi permanent damage*, and *earthquake*.

As shown in Figure 11, the labeled signals could be separated based on the different principal components, which captured varying frequency contributions, as seen in Figure 10b. We now examine the dominant spectral features of some of the labeled categories—*earthquakes*, *vehicles*, and *semi permanent damage*—by combining all the spectral features for each category and plotting the normalized power spectral density versus the frequency. Figure 14 illustrates this, providing insight into why some signals are easily distinguishable at certain frequencies. The *earthquake* spectral features were strongly dominated by low-frequency components at between 1–8 Hz, peaking at 4 Hz. The *semi permanent damage* signals were predominantly characterized by low-frequency features at <3 Hz, which could make them harder to distinguish from other low-frequency signals such as *earthquake* signals. This is also evident looking at Figure 11, where the signals from these sources are difficult, but not impossible, to separate. The *vehicle* spectral features were the most prominent at around 18 and 28 Hz, as further emphasized in Figure 11. The vehicles were clearly isolated into clusters when represented by the second principal component capturing frequencies of around 30 Hz, but these clusters were less distinct when they were represented by the first and third components.

## 4. Discussion and Conclusions

The goal of this study was to develop an automated approach for detecting signals in DAS data, reducing the reliance on manual signal identification. Additionally, the study aimed to analyze the spectral features of detected signals, enabling the separation of different sources in two dimensions using principal component analysis. After creating a labeled dataset, it was used to demonstrate that unlabeled data can be classified using the HDBSCAN algorithm by assigning signals to clusters associated with labeled data. The automated change detection algorithm was found to be fast and efficient, successfully capturing relevant signals with minimal noise, i.e., instances without any signals. It was also developed to be cable-agnostic, meaning that this methodology works on any cable, provided there are enough data to generate a normal representation of the data. However, the methodology for creating bounding boxes around the detected signals was found to be poorly suited for certain signal types, i.e., those of *vehicles*, as seen in Figure 8d–f. This limitation is further highlighted in Figure 9, which shows examples of unlabeled detected signals. In these examples, the signals are not consistently centered in the bounding boxes, which often include substantial non-signal data. This problem directly results from the criteria established in Section 2.2.1 for the creation of bounding boxes. The criterion values were empirically optimized using signals from *ships* and *semi permanent damage*, which makes them less effective for smaller signals. An adaptive approach to the creation of bounding boxes is recommended to address this limitation. This improvement would likely improve the signal separation seen in Figure 12, ensuring that each bounding box only captures a unique signal. In most cases involving labeled *vehicle* signals, as shown in Figure 8, bounding boxes are not practical. It becomes difficult to analyze specific vehicles, because the signals are so frequent that new signals appear before the previous signals end, leading to overlapping bounding boxes. A more effective method would involve extracting pixel values from the binary mask and applying connected-component labeling to link neighboring pixels. Inaccuracies in the bounding boxes for certain signals also impacted the analysis of individual principal components, as demonstrated in Figure 10. Only 90% of the information from the spectral features was contained in the first three principal components, potentially because the PSD used for the PCA contained too many overlapping signals. Addressing this issue through more accurate bounding box creation and improved signal extraction methods would likely enhance the effectiveness of the analysis. Examples of misclassified or ambiguously clustered signals are visible in Figure 12, where bounding boxes containing multiple short-duration vehicle signals skewed the clustering performance. In such cases, the algorithm could not reliably isolate distinct sources, resulting in incorrect or inconsistent cluster assignments, leading to two different clusters for the *vehicle* category. Additionally, cluster 5 exhibited large intra-cluster dispersion, which may reflect the heterogeneous nature of the underlying signal sources. While it was labeled as *semi permanent damage*, it is likely that this cluster contained a mixture of persistent low-frequency signals caused by environmental and infrastructural phenomena. Possible contributors include cable strumming induced by current or flow interactions, tidal harmonics related to cyclic ocean pressure variations, and other long-duration or repeating events. These environmental changes, though not necessarily indicative of structural damage, can produce signals with similar spectral content and temporal characteristics, complicating clustering and interpretation. A more detailed environmental analysis and incorporation of auxiliary oceanographic or cable movement data could help disambiguate such signals in future work.

This study demonstrates that the resolution of the classification method is fundamentally limited by how well the signals are separated in terms of both time and frequency. When bounding boxes overlap or contain multiple distinct events, the resulting features become mixed, reducing the discriminative power of the PCA and degrading the performance of the clustering algorithm. To address these limitations, several mitigation strategies are proposed. First, instead of using fixed-size or threshold-based criteria, an adaptive windowing approach could dynamically adjust the size of bounding boxes based on the local signal density or variance. Second, connected-component labeling could be used on the binary mask to extract contiguous regions of activity, allowing for more precise bounding of individual signals. This method would be particularly effective for high-frequency signal regions such as road vehicle crossings, where signal overlap is frequent. Lastly, allowing for overlapping signal segmentation, where each signal is treated independently even if it partially overlaps with others, could help ensure better feature extraction and classification accuracy.

This study demonstrates the effective detection of signals in DAS using simple methods. The methodology developed allows for near-real-time detection and the possibility of monitoring all the signal types near optic fiber cable networks. This is particularly valuable for monitoring large vast areas with poor coverage from other sensor types, e.g., coastal radars. The current DAS technology limits the sensing range along the cable to approximately 171 km, but ongoing research shows the potential to extend this range even further. We hypothesize, with good-enough methods, that it is possible to distinguish not only between signals from different sources but also different signals originating from the same source, i.e., to tell the difference between individual vehicles.

This study reveals some limitations of conventional methods for signal detection and classification. Improved techniques are necessary to better distinguish signals based on their spectral features, as the PCA approach demonstrated some constraints. In particular, the first principal component captured a broad frequency range, limiting the ability to distinguish between signals above 50 Hz. However, this study still demonstrates that simple and conventional methods can provide enough knowledge for classification and provide valuable insight into the spectral characteristics of different signals. Moreover, the evaluation of the clustering results using the confusion matrix and F1 score metrics confirmed the effectiveness of this classification approach. It achieved high F1 scores across all the signal types, ranging from approximately 0.91 to 0.97. This robust performance suggests that even with relatively simple clustering and classification techniques, the methodology provides reliable signal discrimination, reinforcing its suitability for use in near-real-time monitoring applications. To investigate the classification further, we applied HDBSCAN clustering and tested its robustness with varying levels of labeled data. The resulting clustering metrics, Davies–Bouldin Index, Silhouette Score, and Calinski–Harabasz Index showed that the clustering performance begins to degrade significantly when more than 20–30% of the labeled data is removed. For example, the Davies–Bouldin Index increased from 0.828 (full dataset) to values of above 1.6 when 20–40% of the data were dropped, indicating weaker cluster separation. Similarly, the Silhouette Score dropped into negative values in that range, and the Calinski–Harabasz Index declined from 189.8 to around 120, confirming the reduced cluster quality. These results highlight that although HDBSCAN is effective, its performance is sensitive to the availability of labeled data, and maintaining sufficient labeled examples is critical for stable and interpretable clustering. In future studies a larger labeled dataset should be used in order to create more well-structured clusters that are more reliable when used in classification.

In hindsight, the large volume of data generated by DAS and the limitations of conventional methods highlight the growing need for AI-driven solutions [29]. Notably, a YOLO-based approach has already shown great promise for use in detecting signals [30]. This study can help decrease the long time required to create labeled datasets for training AI models and facilitate easy classification of different signals.

## Figures and Tables

**Table 1:** Number of signals with each label in the dataset.

Label	Count
AIS (Ship)	41
Vehicle	41
semi permanent Damage	77
Earthquake	5
Unlabeled	1312
Total	1476

**Table 2:** F1 score and precision for the classification of labeled signals.

Label	Cluster(s)	TP	FP	FN	Precision	Recall	F1 Score
AIS	4	37	0	2	1.000	0.949	0.974
Earthquake	1	5	1	0	0.833	1.000	0.909
semi permanent damage	5	65	0	12	1.000	0.844	0.915
Vehicle	6, 7	36	0	5	1.000	0.878	0.935

**Table 3:** Clustering metrics for different levels of dropping labeled data.

Drop (%)	Clusters	Davies–Bouldin	Silhouette	Calinski–Harabasz
0	7	0.828	0.124	189.8
10	7	0.833	0.131	189.2
20	3	1.627	−0.047	119.8
30	3	1.587	−0.047	120.1
40	3	1.616	−0.039	119.6
50	4	1.176	0.063	208.8
60	3	1.808	−0.054	110.4
70	4	1.060	0.078	217.5
80	4	1.082	0.073	203.1
90	5	1.018	0.009	146.6

## Data Availability

The data used in this article can be available upon request.
